# Supplementary material for: HIV RNA/DNA Levels at Diagnosis Can Predict Immune Reconstitution: A Longitudinal Analysis
Source: Microorganisms. 2023 Jun 6;11(6):1510. doi: 10.3390/microorganisms11061510 (PMC10300907; doi:10.3390/microorganisms11061510)
Supplement: Supplementary file 1 [file microorganisms-11-01510-s001.zip › microorganisms-2399752-supplementary.pdf]

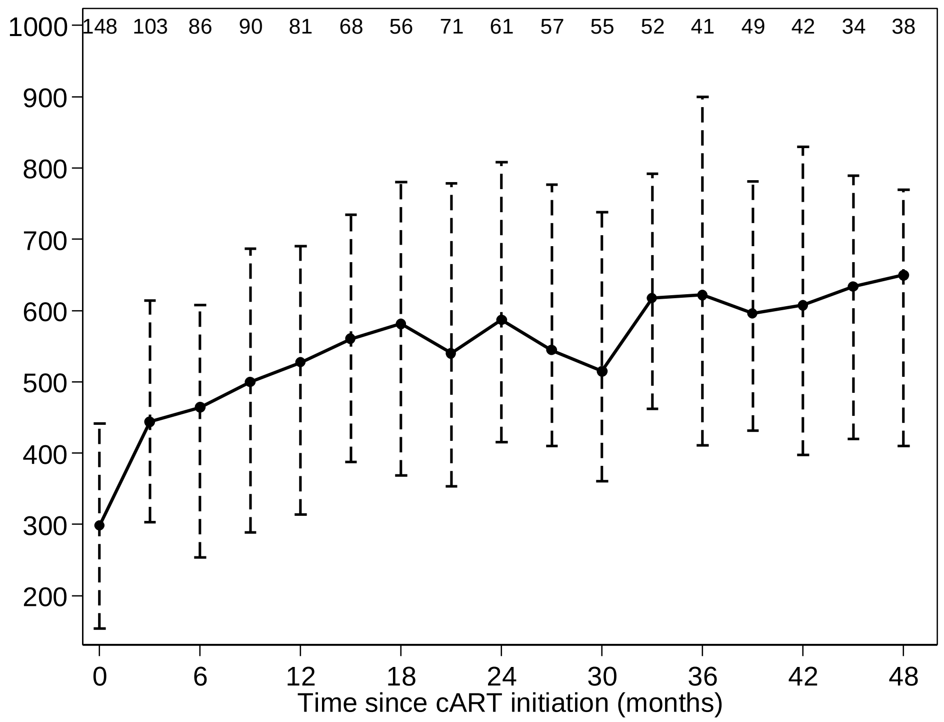

Supplementary Figure S1: CD4 cell count distribution after cART initiation: cross-sectional medians and interquartile ranges (IQR). Figures on top of graph indicate number of patients contributing measurements for each time point.

Supplementary Table S1: Results from a mixed model for CD4 cell count changes after cART according to baseline HIV RNA levels

Factor	Estimate (cells/µL)	95% C.I.	p-value
**CD4 Change 0-3 months**			
per month	60.8	(49.0, 72.5)	**<0.001**
Baseline HIV RNA and CD4 Change 0-3 months interaction			
Above vs. Below median	15.1	(-1.4, 31.5)	0.073
**CD4 Change 3+ months**			
per month	3.2	(2.3, 4.2)	**<0.001**
Baseline HIV RNA and CD4 Change 3+ months interaction			
Above vs. Below median	0.9	(-0.4, 2.3)	0.170

p-values in bold indicate statistical significance

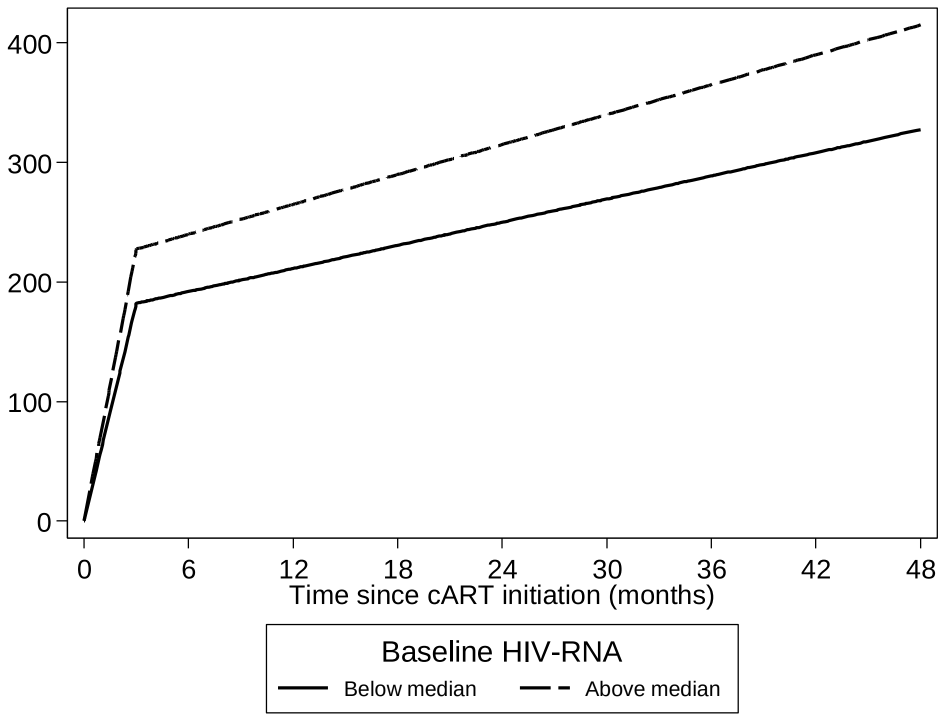

Supplementary Figure S2: Predictions of average CD4 cell count changes based on an unadjusted mixed model for the effects of baseline HIV-RNA

Supplementary Table S2: Results from a mixed model for CD4 cell count changes after cART according to baseline HIV-DNA

**Factor**	**Estimate (cells/µL)**	**95% C.I.**	**p-value**
**CD4 Change 0-3 months**			
per month	68.4	(56.7, 80.1)	**<0.001**
Baseline HIV DNA and CD4 Change 0-3 months interaction			
Above vs. Below median	-0.4	(-17.1, 16.2)	0.960
**CD4 Change 3+ months**			
per month	3.1	(2.2, 4.0)	**<0.001**
Baseline HIV DNA and CD4 Change 3+ months interaction			
Above vs. Below median	1.2	(-0.1, 2.6)	0.071

p-values in bold indicate statistical significance

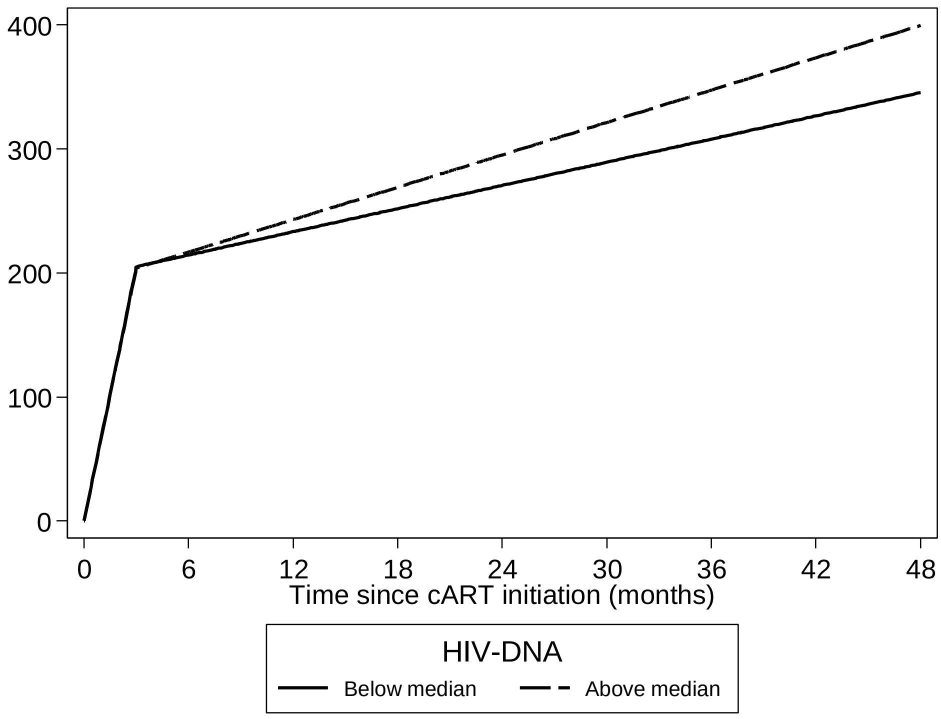

Supplementary Figure S3: Predictions of average CD4 cell count changes based on an unadjusted mixed model for the effects of baseline HIV-DNA

Supplementary Table S3: Results from a mixed model for CD4 cell count changes after cART according to risk group

Factor	Estimate (cells/μL)	95% C.I.	p-value
**CD4 Change 0-3 months**			
per month	76.8	(66.1, 87.5)	**<0.001**
Risk group and CD4 Change 0-3 months interaction			
PWID vs. MSM	-29.8	(-49.2, -10.4)	**0.003**
Heterosexual vs. MSM	-4.5	(-26.6, 17.5)	0.686
**CD4 Change 3+ months**			
per month	3.7	(2.8, 4.5)	**<0.001**
Risk group and CD4 Change 3+ months interaction			
PWID vs. MSM	0.3	(-1.5, 2.0)	0.755
Heterosexual vs. MSM	-0.0	(-1.8, 1.8)	0.993

p-values in bold indicate statistical significance. PWID: people who inject drugs, MSM: men who have sex with men.

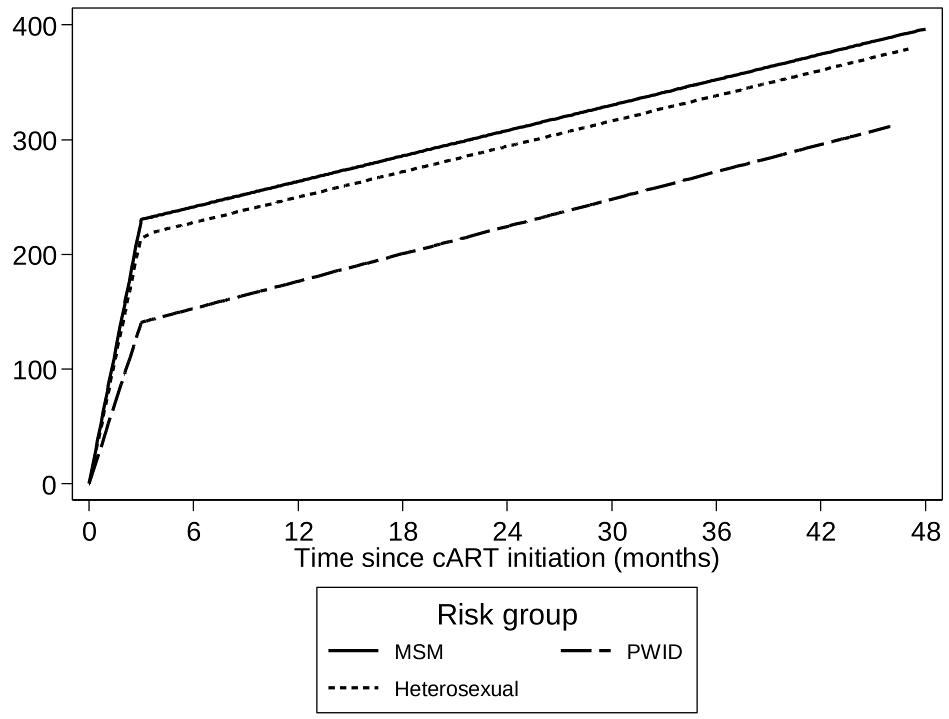

Supplementary Figure S4: Predictions of average CD4 cell count changes based on an unadjusted mixed model for the effects of risk group. PWID: people who inject drugs, MSM: men who have sex with men.

Supplementary Table S4: Results from a multivariable logistic regression model for the risk of suboptimal CD4 response defined as gaining less than 100 CD4 cells/µl during the first 6 months of cART

**Factor**	**Odds Ratio**	**95% C.I.**	**p-value**
Baseline HIV-DNA/RNA combination			
*DNA/RNA: low/low**	1		
DNA/RNA: low/high	0.46	(0.12, 1.74)	0.252
DNA/RNA: high/low	1.30	(0.42, 4.03)	0.648
DNA/RNA:high/high	0.94	(0.28, 3.10)	0.917
Baseline CD4 (cells/microL)			
*0-99**	1		
100-199	0.04	(0.01, 0.29)	**0.001**
200-349	0.02	(0.00, 0.13)	**<0.001**
350-499	0.02	(0.00, 0.19)	**<0.001**
500+	0.06	(0.01, 0.46)	**0.007**
Risk group			
*Non-PWID**	1		
PWID	2.44	(0.95, 6.30)	0.065
Age at cART initiation			
*20-29**	1		
30-39	3.08	(0.96, 9.84)	0.058
40-49	1.11	(0.29, 4.28)	0.874
50+	4.46	(1.07, 18.64)	**0.041**
Type of cART			
*NNRTI**	1		
Boosted PI	0.19	(0.04, 0.99)	**0.049**
INSTI	0.75	(0.18, 3.19)	0.698

* Reference category, PWID: people who inject drugs, cART: combined antiretroviral treatment, PI: protease inhibitor, NNRTI: non-nucleoside reverse transcriptase inhibitor, INSTI: integrase strand transfer inhibitor. P-values in bold indicate statistical significance

Supplementary Table S5: Results from a multivariable logistic regression model for the risk of suboptimal CD4 response defined as having less than 500 CD4 cells/µl at 24 months after cART initiation

**Factor**	**Odds Ratio**	**95% C.I.**	**p-value**
Baseline HIV-DNA/RNA combination			
*DNA/RNA: low/low**	1		
DNA/RNA: low/high	0.52	(0.10, 2.79)	0.449
DNA/RNA: high/low	2.87	(0.75, 10.92)	0.122
DNA/RNA:high/high	0.75	(0.18, 3.10)	0.694
Baseline CD4 (cells/microL)			
*0-99**	1		
100-199	0.21	(0.03, 1.74)	0.149
200-349	0.03	(0.00, 0.23)	**0.001**
350-499	0.02	(0.00, 0.21)	**0.001**
500+	-		
Risk group			
*Non-PWID**	1		
PWID	4.87	(1.48, 16.06)	**0.009**
Age at cART initiation			
*20-29**	1		
30-39	1.26	(0.30, 5.38)	0.753
40-49	2.70	(0.52, 13.88)	0.235
50+	9.77	(1.57, 60.93)	**0.015**
Type of cART			
*NNRTI**	1		
Boosted PI	1.80	(0.40, 7.99)	0.441
INSTI	1.15	(0.16, 8.07)	0.889

* Reference category, PWID: people who inject drugs, cART: combined antiretroviral treatment, PI: protease inhibitor, NNRTI: non-nucleoside reverse transcriptase inhibitor, INSTI: integrase strand transfer inhibitor. P-values in bold indicate statistical significance
